# Supplementary material for: Animal-appropriate housing of ball pythons (Python regius)—Behavior-based evaluation of two types of housing systems
Source: PLoS One. 2021 May 27;16(5):e0247082. doi: 10.1371/journal.pone.0247082 (PMC8158952; doi:10.1371/journal.pone.0247082)
Supplement: Attachment — Submitted filename: Response Reviewer 2.docx [file pone.0247082.s001.docx]

**Manuscript: PONE-D-21-03078**

**Response to reviewer 2**

Dear Reviewer 2

Thank you for reviewing the paper „Animal-appropriate housing of ball pythons (*Python regius*) — Behavior-based evaluation of two types of housing systems”. We appreciate the time and effort that you dedicated to providing feedback on our manuscript. The page and line numbers refer to the amended manuscript.

**Reviewers Comment to the Author**

*I find the work very interesting and important from the point of view of the breeder, caretakers and veterinarians. The discussed topic explains the differences in the maintenance of snakes of this species and also brings many cognitive aspects to their behavioral needs.*

Thank you!

To support the importance of the study, we have added animal numbers (Page 22-23, 459-500). Unfortunately, a number of animals cannot be determined more precisely due to the specific regulations of the countries. We assume a much higher number of unreported cases than in the presented studies, because the surveys could only be conducted with a part of the population and there is, for example, an exception to the obligation to report despite protection status (see Germany).
